# miR-489-3p inhibits proliferation and migration of bladder cancer cells through downregulation of histone deacetylase 2

**DOI:** 10.3892/ol.2020.11869

**Published:** 2020-07-15

**Authors:** Dan Sun, Tianren Li, Haotian Xin, Jun An, Jieping Yang, Jiaxing Lin, Xin Meng, Biao Wang, Toshinori Ozaki, Meng Yu, Yuyan Zhu

**Affiliations:** 1 Department of Urology, The First Hospital of China Medical University, Shenyang, Liaoning 110001, P.R. China; 2 Department of Gynecology, The First Hospital of China Medical University, Shenyang, Liaoning 110001, P.R. China; 3 Department of Biochemistry and Molecular Biology, School of Life Sciences, China Medical University, Shenyang, Liaoning 110122, P.R. China; 4 Department of DNA Damage Signaling, Research Center, The 5th Hospital of Xiamen, Xiamen, Fujian 361101, P.R. China; 5 Key Laboratory of Transgenetic Animal Research, Department of Laboratory Animal Science, China Medical University, Shenyang, Liaoning 110122, P.R. China

**Keywords:** microRNA-489-3p, HDAC2, BC, progression

## Abstract

Since human bladder cancer (BC) is a common malignancy of the urinary system with poor prognosis, it is crucial to clarify the molecular mechanisms of BC development and progression. To the best of our knowledge, the current study demonstrated for the first time that miR-489-3p suppressed BC cell-derived tumor growth *in vivo* via the downregulation of histone deacetylase 2 (HDAC2). According to the results, expression levels of miR-489-3p were lower in BC tissues compared with corresponding normal tissues. Expression of miR-489-3p mimics in BC-derived T24 and 5637 cells resulted in a significant reduction in proliferation and migration rates. Furthermore, bioinformatics analyses indicated that HDAC2 may be a potential downstream target of miR-489-3p. In contrast to miR-489-3p, HDAC2 was expressed at higher levels in BC tissues compared with corresponding normal tissues. Additionally, small interfering RNA-mediated knockdown of HDAC2 caused a marked decrease in the proliferation and migration rates of T24 and 5637 cells. Consistent with these observations, expression of miR-489-3p mimics attenuated the growth of xenograft tumors arising from T24 cells and resulted in HDAC2 downregulation. In conclusion, the results of the current study indicated that the miR-489-3p/HDAC2 axis serves a role in the development and/or the progression of BC and may be a potential molecular target for the development of a novel strategy to treat patients with BC.

## Introduction

Bladder cancer (BC) is the most frequently diagnosed malignancy of the urinary system. Among all types of cancer, BC is the 10th most common cancer worldwide with an estimated 549,000 new cases and 200,000 deaths in 2018 (1). Furthermore, >70% of newly diagnosed patients with BC exhibit non-muscle invasive BC and ~50–70% of these cases eventually exhibit invasive potential with more aggressive characteristics (2). The standard treatment choice for patients with BC is primarily surgical resection. Patients with BC undergoing surgery are at high-risk of the recurrence, as well as occasional stage progression. In advanced BC, following radical surgery or radiotherapy, patients still have poor outcomes (3). The anticancer drug cisplatin frequently serves as first-line chemotherapy. However, the therapeutic effects in patients with BC remain poor (4). It is crucial to elucidate the molecular mechanisms of the malignant properties of BC to increase the survival times of patients and provide novel strategies and/or molecular targets for the early diagnosis and more effective treatment of patients with BC.

MicroRNAs (miRNAs or miRs) are short non-coding RNAs (18–25 nucleotides in length) that bind to the 3′-untranslated regions (UTRs) of the mRNAs of their target genes in a sequence-specific manner, promoting post-transcriptional inhibition or degradation (5–7). Increasing evidence has indicated that miRNAs serve pivotal roles in the regulation of numerous cellular processes including proliferation, differentiation and development (8–10). Furthermore, miRNAs have been demonstrated to be frequently dysregulated in a variety of human cancers, indicated that miRNAs may tightly participate in the development and/or progression of cancer (11,12). For example, it has been described that miR-221 is aberrantly overexpressed in breast cancer and has an ability to stimulate the migration/invasion of breast cancer cells (13). Liang *et al* (14) reported that colon cancer tissues expressed lower levels of miR-141-3p compared with corresponding normal tissues and that the overexpression of miR-141-3p in colon cancer cells attenuated proliferation, migration and invasion rates. These findings indicated that miRNAs may act as oncogenes and/or tumor suppressor genes.

Schoolmeesters *et al* (15) demonstrated that miR-489 served a critical role in the regulation of osteogenesis. Cheung *et al* (16) revealed that miR-489 was implicated in mammalian stem cell proliferation. Similar to other miRNAs, such as miR-26 and let-7 (17,18), miR-489 may also be involved in carcinogenesis. Zhang *et al* (19) demonstrated that miR-489 was downregulated in gastric cancer tissues compared with corresponding normal tissues and overexpression of miR-489 in gastric cancer cells suppressed proliferation and invasion. Yuan *et al* (20) reported that miR-489 acted as an inhibitor of pancreatic cancer invasion. Additionally, Gao *et al* (21) revealed that the elevated expression level of miR-489 was significantly associated with a prolonged survival rate of patients with colon cancer and overexpression of miR-489 in colon cancer cells inhibited their migration and invasion abilities. These observations indicated that miR-489 may act as a tumor-suppressor in a cancer type-independent manner.

The current study focused on miR-489-3p and investigated its functional role in BC. According to the results, there was an inverse relationship between the expression levels of miR-489-3p and its downstream target histone deacetylase 2 (HDAC2) in BC tumor tissues. Depletion of miR-489-3p and HDAC2 increased and decreased the proliferation and migration abilities of BC cells, respectively. Consistent with these observations, increased expression of miR-489-3p suppressed *in vivo* tumor growth and markedly reduced HDAC2 expression. In conclusion, the results of the current strongly indicated that the miR-489-3p/HDAC2 axis serves a vital role in the regulation of the development and/or progression of BC.

## Materials and methods

### 

#### Cells and culture

Human BC-derived T24 and 5637 cells and 293T cells were obtained from The Cell Bank of Type Culture Collection of the Chinese Academy of Sciences. Cells were maintained in RPMI 1640 medium (Gibco; Thermo Fisher Scientific, Inc.) supplemented with 10% heat-inactivated FBS (Gibco; Thermo Fisher Scientific, Inc.). Cells were cultured in incubators with humidified atmospheres of 5% CO_2_ and 95% air at 37°C.

#### Transfection

T24 and 5637 cells were seeded in six-well plates at the density of 0.5×10^6^ cells/well and were transfected using Lipofectamine^®^ 2000 (Invitrogen; Thermo Fisher Scientific, Inc.), according to the manufacturer's protocol. The double-stranded miR-389-3p mimics, agomir and corresponding negative control (NC) RNAs (Suzhou GenePharma Co., Ltd.) or their inhibitors and antagomir were introduced into cells at a final concentration of 50 nM. At 48 h post-transfection, cells were collected for further experiments. The sequence of miR-489-3p mimics and its inhibitor were as follows: miR-489-3p mimics/agomirs forward, 5′-GUGACAUCACAUAUACGGCAGC-3′ and reverse, 5′-UGCCGUAUAUGUGAUGUCACUU-3′; negative control forward, 5′-UUCUCCGAACGUGUCACGUTT-3′ and reverse, 5′-ACGUGACACGUUCGGAGAATT-3′; miR-489-3p inhibitor/antagomir, 5′-GCUGCCGUAUAUGUGAUGUCAC-3′; and miR-489-3p inhibitor/agomir negative control, 5′-CAGUACUUUUGUGUAGUACAA-3′. Negative control siRNA and siRNA against histone deacetylase 2 (cat. no. sc-44262; Santa Cruz Biotechnology, Inc.) were introduced into T24 and 5637 cells at a final concentration of 10 nM. miR-489-3p inhibitor and siRNA against HDAC2 were simultaneously introduced into T24 cells at final concentrations of 50 and 10 nM, respectively, for a duration of 48 h. Empty lentivirus (Lv-NC) vector and miR-489-3p lentivirus expressing vector (Lv-miR-489-3p, H1-has-miR-489-3p-CMV-GFP-puro) were purchased from Suzhou GenePharma Co., Ltd.. To obtain miR-489-3p-overexpressing T24 cells, 5 µl of the miR-489-3p lentivirus expressing vector solution (virus titer=5×10^8^ TU/ml) with 5 µg/ml polybrene were added to T24 cells (MOI=10). Puromycin (1 µg/ml) was used to select transfected cells. The concentration of puromycin used for transfection maintenance was also 1 µg/ml. At 72 h post-transfection, miR-489-3p expression levels were determined by reverse transcription-quantitative PCR (RT-qPCR).

#### Patients and tissue samples

The 31 BC tissues and 11 corresponding adjacent non-cancer tissues used in the present study were obtained from 31 patients who were pathologically diagnosed with urothelial BC and who underwent transurethral bladder tumor resection (20 cases) or radical cystectomy (11 cases) between January, 2014 and February 2017 at the First Hospital of China Medical University (Shenyang, China). The average age of patients was 70.6 years (age range, 61–80 years) and the sex distribution was 24 males and 7 females. The recruitment lasted for 30 months. The freshly collected tissues were fixed with 10% formalin at room temperature for 24 h, frozen in liquid nitrogen and stored at −80°C for histological examination and RT-qPCR. The adjacent normal non-cancerous tissues were collected at locations >5 cm from tumors. All tissues were histologically examined by two pathologists. The present study was approved by the Research Ethics Committee of China Medical University and written informed consent was obtained from all patients.

#### In vivo animal studies

Tumor-formation experiments in nude mice were performed at the Experimental Animal Center of China Medical University. The animal study was approved by Institutional Animal Care and Use Committee of China Medical University (approval no. 2018160). A total of 10 BALB/C nude female mice (body weights, 14–16 g; age, 6 weeks) were obtained from Charles River Laboratories, Inc.. Mice were randomly divided into 2 groups randomly: Lv-NC or Lv-miR-489-3p. Each group contained 5 mice, which is experimentally estimated to be able to satisfactorily detect the growing difference of xenografted tumors between two groups (22). T24 cells were infected with Lv-miR-489-3p or Lv-NC, according to the manufacturer's protocol. A total of 1×10^7^ T24 cells transfected with Lv-NC or Lv-miR-489-3p were subcutaneously implanted into the right axilla of the mice. All mice were housed and maintained under specific pathogen-free conditions in clear cages with free access to food and water at room temperature (22–25°C) and 50% humidity with 12-h light/dark cycles. Animal health and behavior were monitored every two days. In the current study, humane endpoints included: i) Tumor size reaching a diameter of 1.5 cm; ii) tumor surface bleeding; and iii) the presence of cachexia. When one of these symptoms appeared in the mice, the experiment was terminated immediately and cervical dislocation was performed. The protocol was scheduled for 28 days. No mice died during the experiment. All mice were anaesthetized with an intraperitoneal injection of 50 mg/kg pentobarbital sodium (Sigma-Aldrich; Merck KGaA) and cervical vertebrae were dislocated. Following euthanasia, lack of heartbeat was used to verify death. The maximum diameter of the observed tumors was 13 mm. No mice had multiple tumors. The maximum percentage of weight loss observed from start to endpoint was 14.29%. Tumor volume was calculated as follows: Volume (mm^3^)=width^2^ (mm^2^) × length (mm) ×0.4.

#### Animal tissue staining

Tumors were immersed in 4% paraformaldehyde at room temperature for 4 h, and transferred to 70% ethanol overnight at 4°C. Then tumors were placed in processing cassettes, dehydrated through an increasing ethanol gradient (70, 80, 90 and 100%), and embedded in paraffin wax blocks. Sections (5-µm-thick) were dewaxed in xylene, rehydrated through decreasing concentrations of ethanol (100, 90, 80 and 70%), and washed in PBS. Then sections were stained with hematoxylin and eosin (H&E). After staining, sections were dehydrated through increasing concentrations of ethanol and xylene and sealed with neutral gum.

#### Bioinformatics analysis

To predict the target genes of has-miR-489-3p, the TargetScan database (www.targetscan.org, Release 7.1) and Oncomir database (www.oncomir.org) were interrogated, The venn diagram was generated by FunRich software (version 3.1.3) to determine the overlapping target genes between TargetScan and Oncomir.

#### Transwell assay

Transwell assays were performed to assess the migration abilities of the transfected T24 and 5637 cells using 6.5 mm Transwell plates with 8.0 µm Pore Polycarbonate Membrane Inserts (cat. no. 3422; Corning, Inc.). Briefly, 1×10^4^ cells were suspended in 200 µl of serum-free RPMI 1640 medium and seeded in the upper chamber. In the lower chamber, the medium was supplemented with 10% heat-inactivated FBS, which was used as a chemoattractant. Cells were incubated in a humidified incubator at 37°C and 5% CO_2_. After 24 h, the non-migrated cells were removed with a cotton tip. The remaining cells on the bottom surface were fixed, stained with 0.1% crystal violet at room temperature for 20 min. Images were captured using a Leica DM3000 microscope (Leica Microsystems GmbH; magnification, ×40 and ×100). The numbers of cells were counted in ≥5 independent fields of view fields using ImageJ 1.51v software (National Institutes of Health).

#### RNA isolation and RT-qPCR

Total RNA, including micro-RNA from cultured cells and frozen bladder tissues, was extracted using a miRNeasy Mini kit (Qiagen; GmbH), according to the manufacturer's protocol. cDNA of the coding genes was synthesized using a Prime Script RT Master Mix kit (Takara Biotechnology Co., Ltd.; cat. no. RR360A) and cDNA of miRNAs was generated using a Mir-XTM miRNA First-Strand Synthesis kit (Takara Biotechnology Co., Ltd.; cat. no. 638313), according to the manufacturer's protocol. PCR reactions were performed using SYBR Premix Ex Taq^™^ kit (cat. no. RR420A) and SYBR Premix Ex Taq™ (cat. no. RR820A; Takara Biotechnology Co., Ltd.) according to the manufacturer's protocol. β-actin and U6 were used as internal controls. The sequences of the primers were as follows: miR-489-3p 5′-GTGACATCACATATACGGCAG-3′; HDAC2 forward, 5′-TGTGAGATTCCCAATGAGTTGC-3′ and reverse, 5′-GGTAACATGCGCAAATTTTCAA-3′; and β-actin forward, 5′-ACTTAGTTGCGTTACACCCTT-3′ and reverse, 5′-GTCACCTTCACCGTTCCA-3′. The primer of miR-489-3p for RT-qPCR was the mRQ 3′ Primer in Mir-XTM miRNA First-Strand Synthesis kit. The forward and reverse primers for U6 were also provided in the kit Mir-XTM miRNA First-Strand Synthesis kit. The thermocycling conditions for miRNA were as follows: 95°C for 10 sec, 95°C for 5 sec, 60°C for 20 sec for 50 cycles, 95°C for 1 min, 40°C for 1 min, 65°C for 1 sec and 40°C for 5 sec. The thermocycling conditions for HDAC2 were as follows: 95°C for 30 sec, 95°C for 5 sec, 60°C for 30 sec for 45 cycles, 95°C for 1 min, 40°C for 1 min, 65°C for 1 sec and 40°C for 5 sec. The relative expression levels were normalized to endogenous control (U6 or β-actin) and were expressed as 2^−ΔΔCq^ (23).

#### Western blotting

T24 and 5637 cells and tumor tissues were lysed using RIPA protein extraction reagent (Beyotime Institute of Biotechnology) supplemented with 1% protease inhibitor cocktails (Roche Applied Science). Protein concentration was measured using the BCA assay (Beyotime Institute of Biotechnology; cat. no. P0012). Equal amounts of proteins (20 µg/lane) were separated by 10% SDS-PAGE and transferred onto PVDF membranes (EMD Millipore). Following blocking with 5% non-fat milk at 4°C overnight, the membranes were probed with rabbit anti-HDAC2 monoclonal antibodies (1:2,000; Cell Signaling Technology, Inc.; cat. no. 57156s), mouse anti-β-actin monoclonal antibodies (1:3,000; Cell Signaling Technology, Inc.; cat. no. 3700), or rabbit anti-GAPDH (1:5,000; cat. no. sc-25778; Santa Cruz Biotechnology, Inc.) antibodies at room temperature for 1 h, followed by incubation with horseradish peroxidase-conjugated secondary antibodies (1:4,000; OriGene Technologies, Inc.; cat. no. ZDR-5307, goat anti-mouse IgG/HRP and cat. no. ZDR-5306, goat anti-rabbit IgG/HRP) at room temperature for 1 h. Enhanced chemiluminescence reagent (Merck KGaA) was used to detect the signal on the membrane (Beijing Transgen Biotech Co., Ltd.).

#### Luciferase reporter assay

293T cells were transfected using Lipofectamine^®^ 2000 (Invitrogen; Thermo Fisher Scientific, Inc.) with a constant amount of luciferase (Luc) reporter (GeneChem Co., Ltd.) constructs containing wild-type HDAC2 3′-untranslated region (3′-UTR) (Luc-HDAC2 3′-UTR) or mutant HDAC2 3′-UTR (Luc-HDAC2 MUT 3′-UTR), *Renilla* luciferase plasmids, miR-489-3p mimics and control mimics. At 24 h post-transfection, cell lysates were prepared and luciferase activities were measured using a Dual-Luciferase reporter assay system (Promega Corporation), according to the manufacturer's protocol.

#### Cell proliferation assay

Proliferation of T24 and 5637 cells was examined using a Cell Counting kit-8 (CCK-8; Nanjing KeyGen Biotech Co., Ltd.). Briefly, the transfected T24 and 5637 cells were seeded into 96-well plates at a density of 1×10^3^ cells/well. At the time points 0, 24, 48, 72, 96 and 120 h following seeding, 10 µl of CCK-8 reagent was added to each well. Cell proliferation values were determined according to the manufacturer's protocol. Experiments were performed in triplicate.

#### Colony formation assay

T24 and 5637 cells were seeded into six-well plates at a density of 1×10^3^ cells/well in RPMI 1640 medium supplemented with 10% heat-inactivated FBS. At 1 week post-seeding, images were acquired using a light microscope (magnification, ×40). The number of viable colonies was defined as >50 cells/colony. Results were quantified using ImageJ 1.51v software (National Institutes of Health).

#### Wound healing assay

T24 cells and 5637 cells were seeded into six-well plates at the density of 6×10^5^/well, maintained at 37°C overnight, and transfected with miR-489-3p mimics or inhibitors as aforementioned. When the culture had reached ~90% confluency, the cell layer was scratched with a sterile plastic tip. The cell layer was then immediately washed twice with PBS and cultured in serum-free RPMI 1640 medium at 37°C. At 0 and 12 h time points following scratch, wound healing was measured. The closure area of wound was calculated as follows: Migration area (%)=(A0-A12)/A0×100, where A0 represents the area of initial wound area and A12 represents the remaining area of wound after 12 h. The areas were quantified using ImageJ 1.51v software.

#### Tissue arrays

Human BC tumor tissue arrays (cat. no. BlaU066Su01) were purchased from Shanghai Outdo Biotech Co., Ltd.. All of the specimens were collected from 56 patients with BC between January 2007 and February 2011. A total of 11 were lost to follow-up and were excluded, resulting in 45 patients with urothelial BC included. The average age of the 45 patients was 70.1 years (age range, 50–85 years), and comprised 37 men and 8 women. All patients underwent transurethral bladder tumor resection or radical cystectomy between May 2007 and January 2011, and the tumor specimens and adjacent specimens were histologically evaluated by two pathologists. A total of 10 specimens from this tissue array had their corresponding normal tissues. Aperio ImageScope software (version no. v12.3.2.8013; Leica Microsystems, Inc.A) was used according to the manufacturer's protocol. Immunostained microarrays were scored by multiplying the intensity (0–3) and extent (0–100) of staining for each tissue sample, as previously described by Bollag *et al* (24). The expression of miR-489-3p and HDAC2 in BC tumor tissues at different clinical stages (1, 2, 3 and 4) was examined.

#### Immunohistochemical staining (IHC)

Briefly, the tissue array blocks were first dewaxed in xylene and rehydrated with graded ethanol solution (100, 90, 80 and 70%), incubated with methyl alcohol containing 3% hydrogen peroxide and immersed in a citrate buffer for antigen retrieval. IHC staining was performed using Streptavidin-Peroxidase IHC assay kit (OriGene Technologies, Inc. cat. no. SP-9001). After blocking with normal goat serum for 15 min at room temperature, the tissue array blocks were incubated with rabbit anti-HDAC2 monoclonal antibody (Cell Signaling Technology, Inc.; cat. no. 57156s; 1:50) at 4°C overnight, then washed with PBS three times for 3 min. After that the tissue array blocks were incubated with biotin-labeled goat anti-rabbit secondary antibody at 37°C for 15 min, sections were washed with PBS three times for 3 min. The tissue array blocks were incubated with streptavidin-biotinylated-complex/horseradish peroxidase at 37°C for 15 min, then washed with PBS three times for 3 min. DAB staining was conducted at room temperature for 3 min, and sections were stained with hematoxylin for 2 min. Then the tissue array blocks were dehydrated with increasing concentrations of ethanol (70, 80, 90 and 100%). After being immersed in dimethylbenzene for 15 min, slides of tissue array blocks was sealed with neutral resin. Immunostaining was evaluated by two pathologists using a blind protocol design.

For *in situ* hybridization (ISH), formalin-fixed paraffin-embedded tissue array blocks with specimens were deparaffinized in xylene, rehydrated with graded ethanol solution (100, 96 and 70%), and digested by proteinase K (5 µg/ml; Beyotime Institute of Biotechnology; cat. no. ST535) for 2 min at 37°C, then dehydrated by increasing concentrations of ethanol (70, 96 and 100%). Then, the tissue array blocks were incubated with miR-489-3p-probe solution (Exiqon; Qiagen; cat. no. 612051-360) for 1 h at 50°C. The tissue array blocks were then washed with graded sodium citrate buffer. After blocking with Roche DIG Wash and Block Buffer (Roche Diagnostics; cat. no. 11585762001) for 15 min at room temperature, the tissue array blocks were incubated with Anti-Digoxigenin-AP, Fab fragments (Roche Diagnostics; Cat. no. 11093274910150U) blocking solution at 4°C overnight. After washing with TBST, the tissue array blocks were stained with NBT/BCIP (Vector Laboratories, Inc.; cat. no. SK-5400) and nuclear fast red dye successively and then sealed with neutral resin. Images were acquired using Leica Aperio Slide Scanner (Leica Microsystems, Inc., magnifications, ×50 and ×200).

#### Statistical analysis

Data are presented as the mean ± standard deviation. Experiments were performed in triplicate. Student's t-test or one-way ANOVA with Tukey's multiple comparisons post hoc test were used to analyze the differences between two groups or multiple groups, respectively. The expression levels between cancer and adjacent samples were analysed using Wilcoxon test. Pearson's correlation analysis was used to determine the correlation between miR-489-3p and HDAC2 mRNA levels in BC tumor tissues. miR-489-3p and HDAC2 mRNA levels and their tissue array expression scores were analyzed using linear regression. Survival curves were calculated using the Kaplan Meier method and log-rank tests were used to examine the differences in survival rates between the two groups. All statistical analyses were performed using SPSS software (version 20.0; IBM Corp.). P<0.05 was considered to indicate a statistically significant difference.

## Results

### 

#### miR-489-3p is significantly downregulated in BC tumor tissues and attenuates the proliferation and migration of BC cells

**Figure 1.**
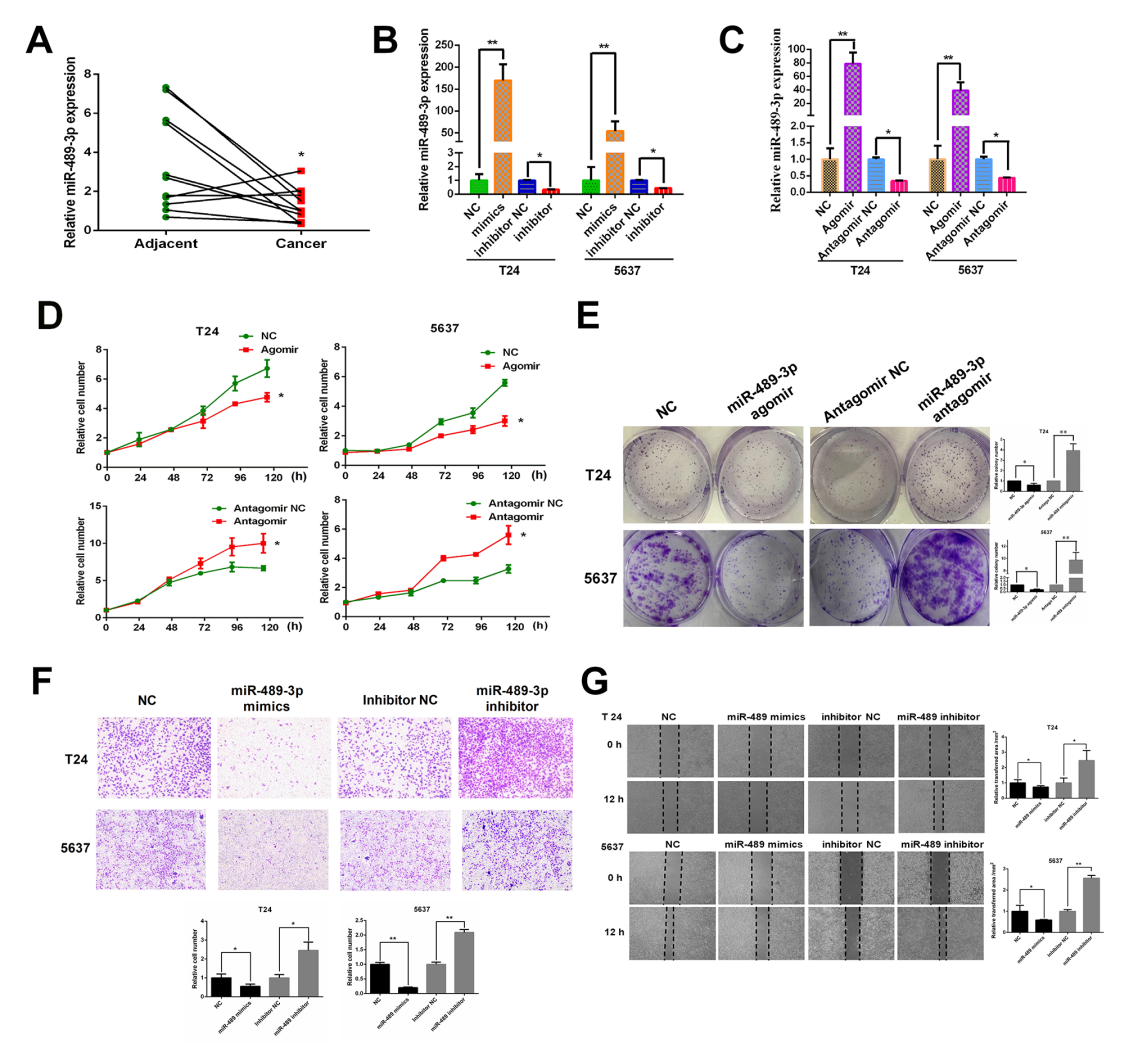
miR-489-3p is expressed at lower levels in BC tissues and attenuates the proliferation and migration ability of BC cells. (A) Lower expression levels of miR-489-3p in BC tissues compared with corresponding normal tissues. A total of 11 pairs of human BC tissues and their corresponding normal tissues were analyzed for miR-489-3p expression by RT-qPCR. *P<0.05 vs. adjacent. (B) Overexpression of miR-489-3p mimics in BC cells. T24 and 5637 cells were transfected with NC, miR-489-3p mimics, inhibitor NC or miR-489-3p inhibitors. At 48 h post-transfection, RT-qPCR was performed to measure miR-489-3p expression. **P<0.01 vs. NC and *P<0.05 vs. inhibitor NC. (C) RT-qPCR was also performed to determine the effect of agomirs and antagomirs. **P<0.01 vs. NC and *P<0.05 vs. antagomir NC. (D) Proliferation rates of cells post-transfection were examined by CCK-8 assay at the indicated time points and the results demonstrated that proliferation rates were reduced by the increased expression of miR-489-3p. *P<0.05 vs. respective NC group. (E) At 48 h post-transfection, cells were maintained in medium. At 1 week post-seeding, the numbers of viable colonies were observed. *P<0.05 and **P<0.01 vs. respective NC group. (F) T24 and 5637 cells were transfected with the indicated mimics or inhibitors and subjected to Transwell assays. The results demonstrated that miR-489-3p inhibited the migration ability of BC cells. Magnification, ×40. *P<0.05 and **P<0.01 vs. respective NC group. (G) Transfected cells were examined using wound healing assays. Magnification, ×40. *P<0.05 and **P<0.01 vs. the respective NC group. miR, microRNA; BC, bladder cancer; RT-qPCR, reverse transcription-quantitative PCR; NC, negative control.

The expression levels of miR-489-3p in 11 BC tumor tissues and their corresponding normal tissues were measured by RT-qPCR to determine the potential role of miR-489-3p in the regulation of human BC. miR-489-3p was expressed at lower levels in BC tumor tissues compared with corresponding adjacent normal tissues, indicating that miR-489-3p may exhibit anti-tumor capabilities against BC (Fig. 1A). To further investigate this, T24 and 5637 cells were transfected with miR-489-3p mimics/agomirs or inhibitors/antagomirs. At 48 h post-transfection, miR-489-3p expression levels were determined by RT-qPCR. miR-489-3p expression levels were significantly increased in T24 cells transfected with miR-489 mimics/agomirs and significantly decreased in cells transfected with miR-489-3p inhibitors/antagomirs compared with their corresponding NCs (Fig. 1B and C). 5367 cells exhibited similar results. Following this, the proliferation rates of the transfected cells were determined. CCK-8 assay demonstrated that the proliferation rates of T24 and 5637 cells were significantly reduced by the increased expression of miR-489-3p and significantly increased by the repression of miR-489-3p compared with the respective NCs (Fig. 1D). In accordance with these results, the colony formation assays revealed that overexpression and repression of miR-489-3p resulted in a decrease and an increase in the number of viable colonies, respectively (Fig. 1E) Furthermore, the effect of miR-489-3p on the migration ability of BC cells was examined. Transwell assays demonstrated that the migration ability of T24 and 5637 cells was significantly decreased and increased by the overexpression and repression of miR-489-3p, respectively (Fig. 1F). Similar results were reported by wound healing assays (Fig. 1G). In summary, the results indicated that miR-489-3p may have a tumor-suppressive role in human BC.

#### HDAC2 is a direct downstream target of miR-489-3p

**Figure 2.**
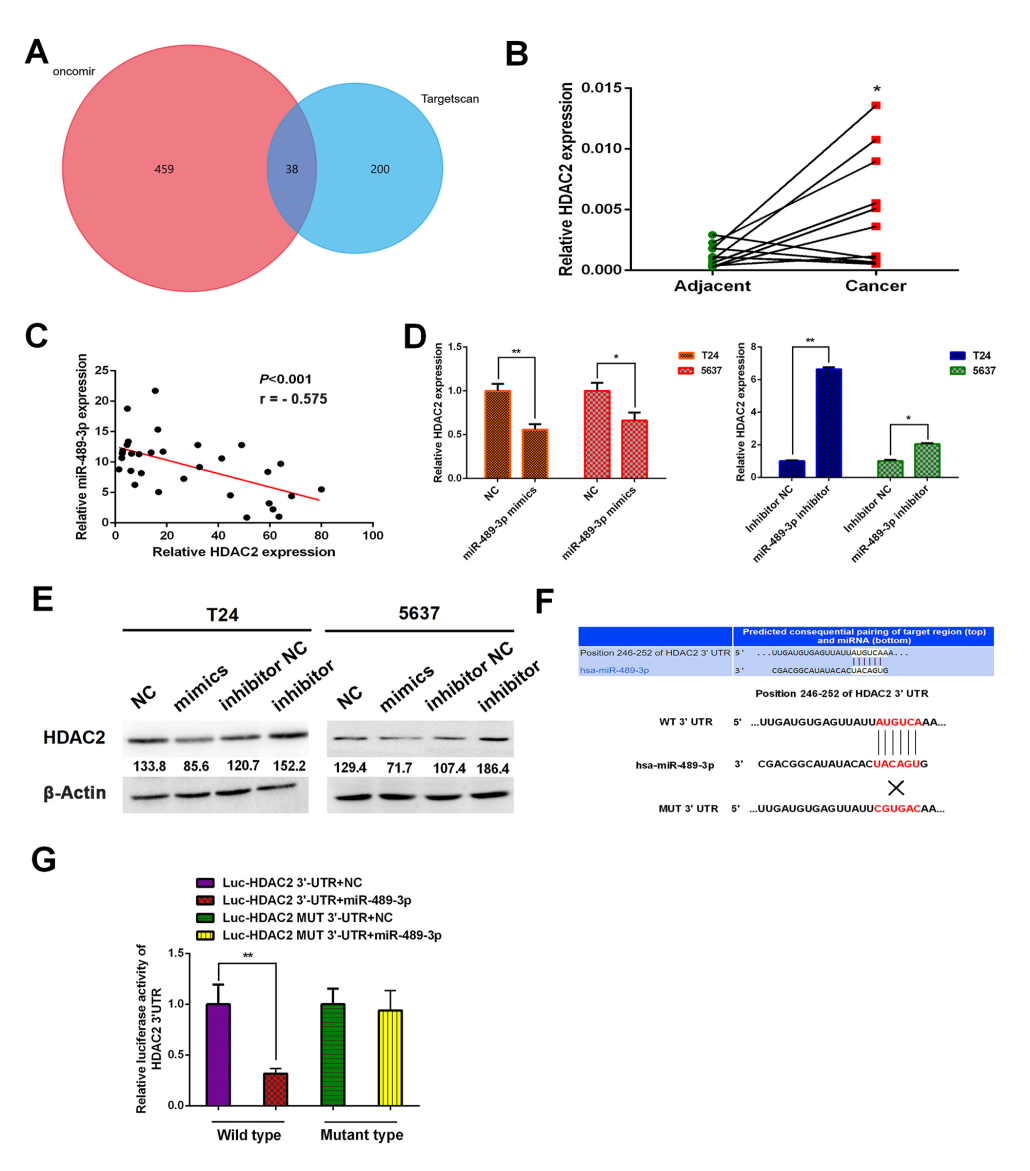
Identification of HDAC2 as a direct target of miR-489-3p in BC cells. (A) Venn diagram demonstrating the numbers of and overlap of the predicted target genes between Oncomir and Targetscan. (B) A total of 11 pairs of human BC tissues and their corresponding adjacent non-cancer ones were analyzed for HDAC2 by RT-qPCR. The results revealed higher expression levels of HDAC2 in BC tissues compared with corresponding normal tissues. *P<0.05 vs. adjacent. (C) Inverse correlation between the expression levels of miR-489-3p and HDAC2 in BC tissues. Pearson's correlation analysis was performed to examine the correlation between the expression levels of miR-489-3p and HDAC2 in BC tissues. T24 and 5637 cells were transfected with the indicated microRNAs and at 48 h post-transfection, total RNA and whole cell lysates were prepared and analyzed by (D) RT-qPCR and (E) western blotting, respectively. Actin was used as the loading control. The results demonstrated that miR-489-3p downregulated HDAC2. (F) 3′-UTR of HDAC2 contains a putative miR-489-3p-binding site as estimated by bioinformatics analysis. The MUT binding sequence is also presented. (G) 293T cells were co-transfected with miR-489-3p mimics or with NC together with Luc-HDAC2 3′-UTR or with Luc-HDAC2 MUT 3′-UTR. Luciferase activity was measured 24 h post-transfection to assess the binding between miR-489-3p and 3′-UTR of HDAC2. *P<0.05 and **P<0.01. HDAC2, histone deacetylase 2; miR/miRNA, microRNA; BC, bladder cancer; RT-qPCR, reverse transcription-quantitative PCR; UTR, untranslated region; NC, negative control; Luc, luciferase; MUT, mutant; WT, wild-type.

To investigate the underlying molecular mechanism of miR-489-3p, putative target genes were identified using two different bioinformatics databases: TargetScan (www.targetscan.org) and Oncomir (www.oncomir.org). The venn diagram was generated by FunRich software (version 3.1.3). There were 38 overlapping target genes between TargetScan and Oncomir. The results indicated that HDAC2 was one of the target genes of miR-489-3p (Fig. 2A). Furthermore, the expression analysis demonstrated that HDAC2 was significantly upregulated in BC tumor tissues compared with corresponding normal tissues (Fig. 2B). The results of Pearson's correlation analysis demonstrated there was an inverse relationship between miR-489-3p expression levels and HDAC2 (Fig. 2C). Following this, the possible effect of miR-489-3p on HDAC2 expression in BC cells was investigated. T24 and 5637 cells were transfected with miR-489-3p mimics or inhibitors. The expression levels of HDAC2 were significantly decreased and increased at the mRNA level in the presence of mimics and inhibitors, respectively (Fig. 2D). Similar results were obtained at the protein level (Fig. 2E). Since HDAC2 3′-UTR contained a putative target site for miR-489-3p (Fig. 2F), luciferase reporter vectors Luc-HDAC2 3′-UTR and Luc-HDAC2 MUT 3′-UTR were constructed. 293T cells were transfected with the aforementioned combinations of vectors and miR-489-3p mimics and the luciferase activities were measured. miR-489-3p mimics significantly suppressed the luciferase activity of the Luc-HDAC2 3′-UTR vector; however, the mimics did not have a significant effect on the Luc-HDAC2 MUT 3′-UTR vector (Fig. 2G). These results indicated that HDAC2 may be a direct downstream target of miR-489-3p.

#### miR-489-3p suppresses the growth of BC tumors in vivo

**Figure 3.**
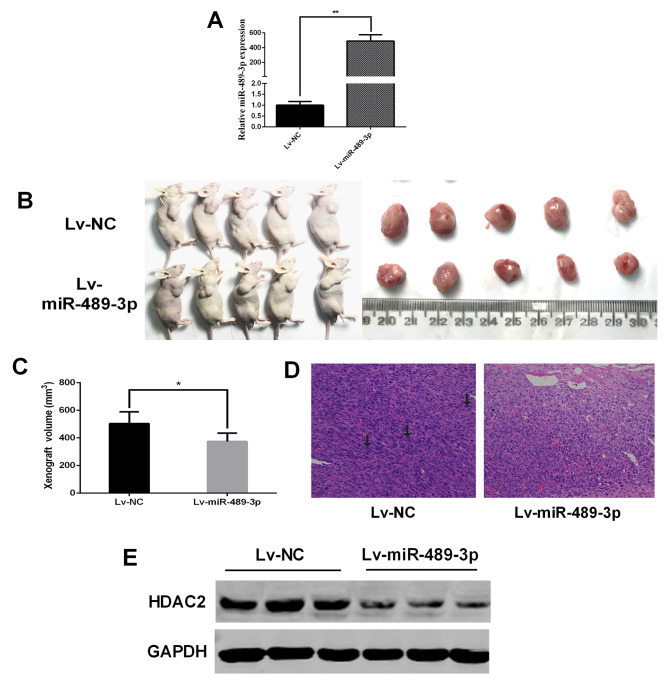
miR-489-3p inhibits bladder cancer tumor growth *in vivo*. T24 cells were transfected with Lv-NC or Lv-miR-489-3p. (A) The expression level of miR-489-3p was determined using reverse transcription-quantitative PCR. The transfected cells were collected and subcutaneously injected into nude mice. At 28 days post-injection, (B) representative images of tumor-bearing BALB/c nude mice and tumors were captured. (C) Tumor volumes were reduced in the Lv-miR-489-3p group. Tumor volume was calculated using the following formula: Volume (mm^3^)=width^2^ (mm^2^) × length (mm) ×0.4. (D) Representative images of hematoxylin and eosin staining of the indicated tumors. Magnification, ×200. (E) HDAC2 expression was decreased in tumors overexpressing miR-489-3p. Whole cell lysates were prepared from the tumors and analyzed for HDAC2 by western blotting. GAPDH was used as the loading control. *P<0.05 and **P<0.01. miR, microRNA; Lv, lentivirus; NC, negative control; Lv-NC, empty lentivirus; HDAC2, histone deacetylase 2; Lv-miR-489-3p, lentivirus expressing miR-489-3p.

*In vivo* xenograft assays were performed to further confirm the tumor-suppressive role of miR-489-3p in BC. T24 cells infected with Lv-NC or Lv-miR-489-3p were introduced subcutaneously into nude mice. RT-qPCR was performed to determine the expression level of miR-489-3p in the transfected cells (Fig. 3A). At 28 days post-injection, a significant decrease in xenograft tumor volumes was observed in the Lv-miR-489-3p group compared with the Lv-NC group (Fig. 3B and C). Histochemical staining demonstrated increased nuclear atypia in the Lv-NC group compared with the Lv-miR-489-3p group (Fig. 3D). Furthermore, western blotting revealed marked HDAC2 downregulation in the tumors in the Lv-miR-489-3p group compared with the Lv-NC group (Fig. 3E). These results indicated that miR-489-3p attenuates *in vivo* BC tumor growth through the downregulation of HDAC2.

#### Depletion of HDAC2 restores the effect of miR-489-3p inhibition on BC cells

**Figure 4.**
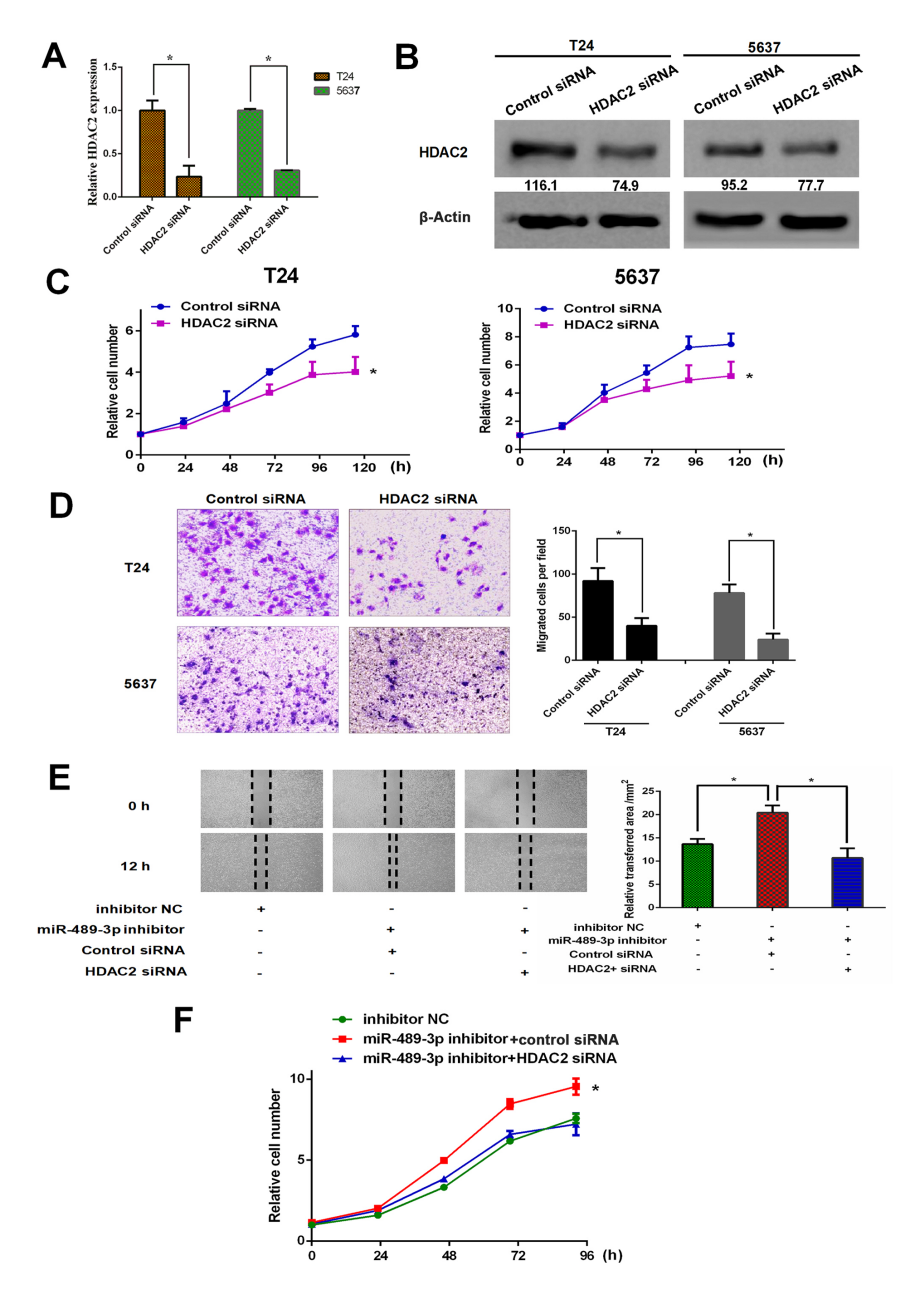
miR-489-3p inhibition-mediated growth promotion is attenuated by HDAC2 knockdown in bladder cancer cells. T24 and 5637 cells were transfected with control siRNA or siRNA against HDAC2. At 48 h post-transfection, total RNA and whole cell lysates were prepared and subjected to (A) reverse transcription-quantitative PCR and (B) western blotting, respectively. Actin was used as a loading control. *P<0.05. (C) T24 and 5637 cells were transfected and cell viability was examined by CCK-8 assays at the indicated time points. *P<0.05 vs. control siRNA. (D) At 48 h post-transfection, the migration ability of the indicated cells was examined by Transwell assays. Representative images were captured and the number of migrated cells was counted. Magnification, ×100. (E) T24 cells were transfected and wound healing assays were performed. Relative wound healing area was calculated. *P<0.05. Magnification, ×40. (F) T24 cells were transfected as in (E). At the indicated time points following transfection, cells were processed for CCK-8 assays. *P<0.05 vs. miR-489-3p inhibitor + HDAC2 siRNA and inhibitor NC. miR, microRNA; HDAC2, histone deacetylase 2; siRNA, small interfering RNA; NC, negative control.

To further address the functional association between miR-489-3p and HDAC2 in BC, HDAC2 gene knockdown in T24 and 5637 cells was performed. siRNA-mediated knockdown of HDAC2 was successful under the experimental conditions (Fig. 4A and B). Following this, the effect of HDAC2 knockdown on the proliferation rates of T24 and 5637 cells was examined. CCK-8 assay demonstrated that the depletion of HDAC2 resulted in a significant reduction in the proliferation rates of both cell lines (Fig. 4C). Furthermore, Transwell assays revealed that the migration abilities of T24 and 5637 cells were significantly reduced by HDAC2 silencing (Fig. 4D). Additionally, T24 cells were simultaneously transfected with miR-489-3p inhibitor and siRNA against HDAC2. The results for the wound healing assays demonstrated that miR-489-3p inhibitor-mediated enhancement of the migration ability of T24 cells was inhibited by the simultaneous depletion of HDAC2 (Fig. 4E). Consistent with these observations, HDAC2 silencing attenuated the miR-489-3p inhibitor-mediated increase in the proliferation rate of T24 cells (Fig. 4F). In summary, these observations indicated that HDAC2, the downstream target of miR-489-3p, has pro-oncogenic potential in BC.

#### Higher expression levels of miR-489-3p and HDAC2 are associated with an improved and poor prognosis in patients with BC, respectively

**Figure 5.**
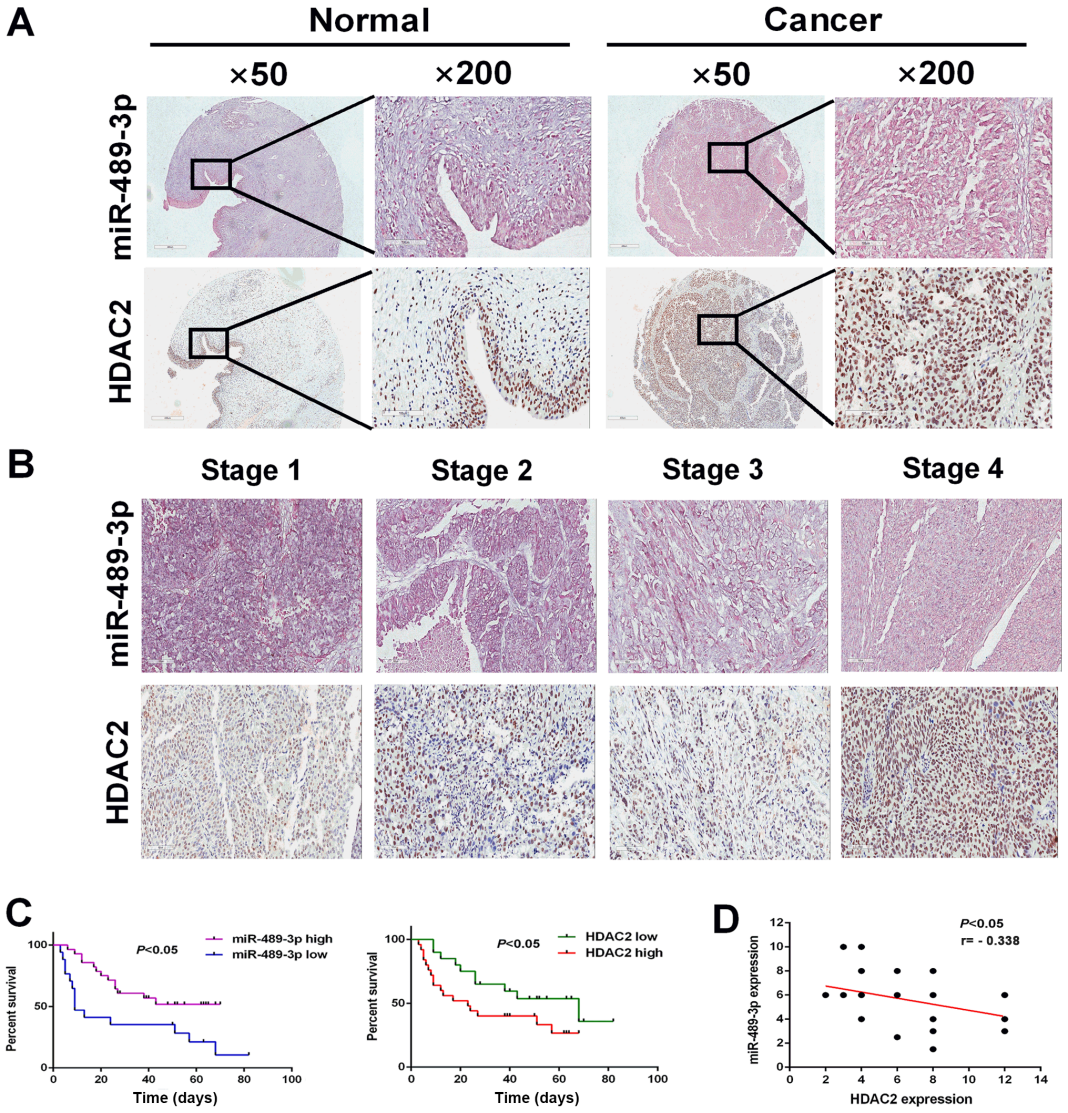
Higher expression levels of miR-489-3p and HDAC2 are associated with an improved and poor prognosis in patients with BC in tissue arrays, respectively. (A) Representative images of miR-489-3p *in situ* hybridization and HDAC2 IHC staining in BC tissues and corresponding normal tissues in tissue microarrays. (B) Representative images of miR-489-3p *in situ* hybridization and HDAC2 IHC staining in BC tissues at different clinical stages in tissue arrays. Magnification, ×200. (C) Kaplan-Meier analysis of the overall survival of 45 patients with urothelial BC with different expression levels of HDAC2 and miR-489-3p. (D) miR-489-3p expression was negatively correlated with HDAC2 expression in urothelial BC tissues. miR, microRNA; HDAC 2, histone deacetylase 2; BC, bladder cancer; IHC, immunohistochemical.

To investigate the possible clinical impact of miR-489-3p and HDAC2 on BC, the expression levels of miR-489-3p and HDAC2 were examined in BC tumor tissues. BC tumor tissues and their corresponding normal tissues were analyzed for miR-489-3p and HDAC2 by *in situ* hybridization and IHC staining. miR-489-3p was expressed at higher level in the corresponding normal tissues compared with BC tumor tissues (Fig. 5A). Furthermore, BC tumor tissues exhibited higher HDAC expression compared with the corresponding normal tissues. Following this, the expression of miR-489-3p and HDAC2 in BC tumor tissues at different clinical stages (1, 2, 3 and 4) was examined. The expression levels of miR-489-3p and HDAC2 were decreased and increased, respectively, in a stage-dependent manner (Fig. 5B). In accordance with these results, Kaplan-Meier analysis demonstrated that the higher expression levels of miR-489-3p (expression score ≥180) and HDAC2 (expression score ≥150) were closely associated with an improved and poor prognosis, respectively, in patients with BC (Fig. 5C). Additionally, there was an inverse relationship between the expression levels of miR-489-3p and HDAC2 in BC tumor tissues (Fig. 5D). These observations indicated that the miR-489-3p/HDAC2 regulatory axis served a role in the development and/or progression of BC.

## Discussion

The results of the current study demonstrated that there was an inverse relationship between the expression levels of miR-489-3p and its direct target HDAC2 in BC, and that the miR-489-3p/HDAC2 axis served a role in the regulation of BC development and/or progression.

To elucidate the functional role of miR-489-3p in BC, its expression levels in BC tumor tissues and corresponding normal tissues were examined. The results revealed that miR-489-3p was expressed at lower levels in BC tumor tissues compared with the corresponding normal tissues, which was consistent with previous observations (25). In addition to BC, miR-489-3p downregulation has been observed in several types of cancer, including colon cancer, breast cancer, ovarian cancer, lung cancer and osteosarcoma, indicating that a decrease in miR-489-3p expression is not restricted to BC (21,26–29). Since downregulated miRNAs have been reported in a number of cancer tissues, they potentially have tumor-suppressive functions (5). Therefore, it is likely that miR-489-3p may act as a tumor-suppressor in BC. siRNA-mediated depletion of miR-489-3p stimulated the proliferation and migration of BC-derived T24 and 5637 cells. Furthermore, the overexpression of miR-489-3p in T24 cells attenuated the *in vivo* xenograft tumor growth. Similarly, Li *et al* (25) reported that the overexpression of miR-489 decreased the proliferation and the invasion rates of BC-derived T24 and UMUC3 cells. Taken together with previous observations, the results of the current study indicated that miR-489-3p exhibits a tumor-suppressive role in BC.

In order to clarify the possible molecular mechanisms by which miR-489-3p suppresses the malignant properties of BC, it is crucial to identify its candidate target genes. Therefore, bioinformatics analysis was performed and HDAC2 was identified as one of the target genes of miR-489-3p. Accumulating evidence has indicated that HDAC2 is involved in DNA damage response, cellular proliferation and apoptosis (30–32). In addition to these cellular processes, HDAC2 has been reported to contribute to carcinogenesis (33). The 3′-UTR of HDAC2 contains a potential target sequence of miR-489-3p and overexpression of miR-489-3p in BC cells downregulated HDAC2 expression. However, Li *et al* (25) demonstrated that jagged canonical notch ligand 1 (JAG1) may be one of the target genes of miR-489. Furthermore, it has been demonstrated that JAG1 was expressed at lower levels in BC tissues compared with corresponding normal tissues, and that patients with BC and lower levels of JAG1 and Notch-1 exhibited shorter survival times (34), indicating that JAG1/Notch-1 signaling pathway may be involved in the suppression of BC. It has been demonstrated that miR-489 reduced the expression level of JAD1; however, the underlying mechanism of miR-489-mediated downregulation of JAG1 in the suppression of BC remains to be elucidated (25).

In contrast to previous reports regarding JAG1, BC tissues used in the current study expressed HDAC2 at higher levels compared with corresponding normal tissues, indicating that there was an inverse relationship between the expression levels of HDAC2 and miR-489-3p in BC. In addition to BC, the abnormal overexpression of HDAC2 has been detected in a number of types of cancer, including gastric cancer, ovarian cancer and breast cancer (35–37). Therefore, it is likely that the dysregulated overexpression of HDAC2 is not restricted to BC. According to the results of the current study, depletion of HDAC2 attenuated the proliferation and migration of BC cells. Moreover, miR-489-3p overexpression-mediated *in vivo* xenograft tumor growth suppression was accompanied by a significant decrease in HDAC2. These observations indicated that, in contrast to miR-489-3p, HDAC2 may have an oncogenic role in BC. Consistent with these results, Niegisch *et al* (38) reported that the expression level of HDAC2 was upregulated in BC cells and Pinkerneil *et al* (39) demonstrated that the double knockdown of HDAC1/HDAC2 inhibited the proliferation of BC cells.

Furthermore, La Noce *et al* demonstrated that HDAC2 gene silencing in osteosarcoma-derived cells promoted cancer stemness and enhanced *in vivo* xenograft tumor growth (40). Liu *et al* (29) reported that miR-489-3p was downregulated in osteosarcoma cells with a high metastatic potential and that miR-489-3p depletion in osteosarcoma cells inhibited metastasis (29). Therefore, it is possible that the pro-oncogenic function of HDAC2 may be dependent on the type of cancer, whereas the tumor-suppressive function of miR-489-3p may not be restricted to a specific cancer type. In addition, it may be hypothesized that HDAC2 could acquire the tumor-suppressive function observed in osteosarcoma. Recently, it has been demonstrated that HDAC2 binds to tumor suppressor p53 and augments its transcriptional activity in p53-wild-type osteosarcoma cells following DNA damage, indicating that the tumor-suppressive activity of HDAC2 may be dependent, at least in part, on p53 (41). Whether the functional conversion of HDAC2 could be dependent on p53, and whether p53 could directly regulate the expression of miR-489-3p, will be further investigated.

The present study demonstrated that HDAC2 may have a dual role in the regulation of carcinogenesis. HDAC2 acted as an oncogenic protein and a tumor-suppressor in bladder cancer and osteosarcoma cells, respectively. How HDAC2 could lose its anti-oncogenic ability and then acquire pro-oncogenic potential in bladder cancer cells remains unknown. In conclusion, the results of the current study indicated that the miR-489-3p/HDAC2 axis is a potential therapeutic target for patients with BC.

## Data Availability

The datasets used and/or analyzed during the current study are available from the corresponding author on reasonable request.

## Abbreviations

HDAC2: histone deacetylase 2
BC: bladder cancer
